# Multiphase CT radiomics nomogram for preoperatively predicting the WHO/ISUP nuclear grade of small (< 4 cm) clear cell renal cell carcinoma

**DOI:** 10.1186/s12885-023-11454-5

**Published:** 2023-10-09

**Authors:** Yankun Gao, Xia Wang, Xiaoying Zhao, Chao Zhu, Cuiping Li, Jianying Li, Xingwang Wu

**Affiliations:** 1 Department of Radiology, The First Affiliated Hospital of Anhui Medical University, Hefei, 230022 Anhui China; 2 CT Research Center, GE Healthcare China, Shanghai, 210000 China

**Keywords:** Clear cell renal cell carcinoma, Small renal mass, Radiomics nomogram, Computed tomography, WHO/ISUP grade

## Abstract

**Background:**

Small (< 4 cm) clear cell renal cell carcinoma (ccRCC) is the most common type of small renal cancer and its prognosis is poor. However, conventional radiological characteristics obtained by computed tomography (CT) are not sufficient to predict the nuclear grade of small ccRCC before surgery.

**Methods:**

A total of 113 patients with histologically confirmed ccRCC were randomly assigned to the training set (n = 67) and the testing set (n = 46). The baseline and CT imaging data of the patients were evaluated statistically to develop a clinical model. A radiomics model was created, and the radiomics score (Rad-score) was calculated by extracting radiomics features from the CT images. Then, a clinical radiomics nomogram was developed using multivariate logistic regression analysis by combining the Rad-score and critical clinical characteristics. The receiver operating characteristic (ROC) curve was used to evaluate the discrimination of small ccRCC in both the training and testing sets.

**Results:**

The radiomics model was constructed using six features obtained from the CT images. The shape and relative enhancement value of the nephrographic phase (REV of the NP) were found to be independent risk factors in the clinical model. The area under the curve (AUC) values for the training and testing sets for the clinical radiomics nomogram were 0.940 and 0.902, respectively. Decision curve analysis (DCA) revealed that the radiomics nomogram model was a better predictor, with the highest degree of coincidence.

**Conclusion:**

The CT-based radiomics nomogram has the potential to be a noninvasive and preoperative method for predicting the WHO/ISUP grade of small ccRCC.

**Supplementary Information:**

The online version contains supplementary material available at 10.1186/s12885-023-11454-5.

## Introduction

The increasing use of cross-sectional imaging in recent decades has led to an increase in the incidence of renal cell carcinoma (RCC) [1]. RCC accounts for approximately 90% of renal tumors, with clear cell renal cell carcinoma (ccRCC) responsible for nearly 80% of cases [2]. A small renal mass (SRM), defined as a tumor less than 4 cm in diameter, constitutes around 40% of all kidney tumor [3, 4]. While most SRMs are malignant, ccRCC remains the most common type of renal malignancy. Despite early detection and resection of RCC, the mortality rate has not decreased significantly, indicating that small RCC is not the primary cause of death from RCC [5, 6]. Several studies have demonstrated that active surveillance of patients with small ccRCC, particularly those with a limited life expectancy or who decline surgical treatment, does not result in a significant increase in mortality [7, 8].

Most small RCCs appear as benign tumors, but some can exhibit high aggressiveness and have the potential to spread to the perirenal fat or distant locations [9, 10]. The World Health Organization/International Society of Urological Pathology (WHO/ISUP) criteria, established in 2016, is the widely accepted classification system for ccRCC. It categorizes ccRCC into four grades, with grades I and II considered low-grade and grades III and IV considered high-grade [11]. High-grade ccRCC is known to be more aggressive, prone to metastasis, and associated with poorer outcomes. Predicting the tumor grade beforehand can aid in determining appropriate treatment strategies. Percutaneous puncture pathology biopsy is a commonly used method for preoperative grading of ccRCC, but it is an invasive procedure. Heterogeneity within the tumor may result in a low histological grade, which can delay treatment [12, 13]. A noninvasive and effective method for determining the histological grade of small ccRCC is necessary.

The most popular noninvasive diagnostic tool for determining whether a small renal mass is benign or malignant, and for identifying the histological grade of small ccRCC, is computed tomography (CT) [14–16]. However, poor interobserver agreement and inconsistent performance make it difficult to use these radiological characteristics in clinical practice. Additionally, there are many overlapping imaging features between high-grade and low-grade tumors [17].

Artificial intelligence is increasingly employed in medical imaging to extract features from medical images that are imperceptible to the naked eye. For instance, radiomics, which is based on CT images, and pathomics, which is based on whole slide images (WSI) [18–21]. Radiomics has been successfully in various areas related to SRM, including differentiating between benign and malignant SRM and grading small RCC [22–26]. Pathomics features can serve as a novel prognostic marker for predicting the prognosis of patients with ccRCC [27]. Most research has focused on textural features, and has not considered the potential value of clinical data and imaging characteristics, which could improve the diagnostic accuracy of the models.

The purpose of this study is to evaluate the ability of multiphase CT-based radiomics nomogram analysis, which combines radiomics features and clinic-radiological characteristics, to predict the WHO/IUSP grade of small ccRCC.

## Patients and methods

### Patients

**Fig. 1:**
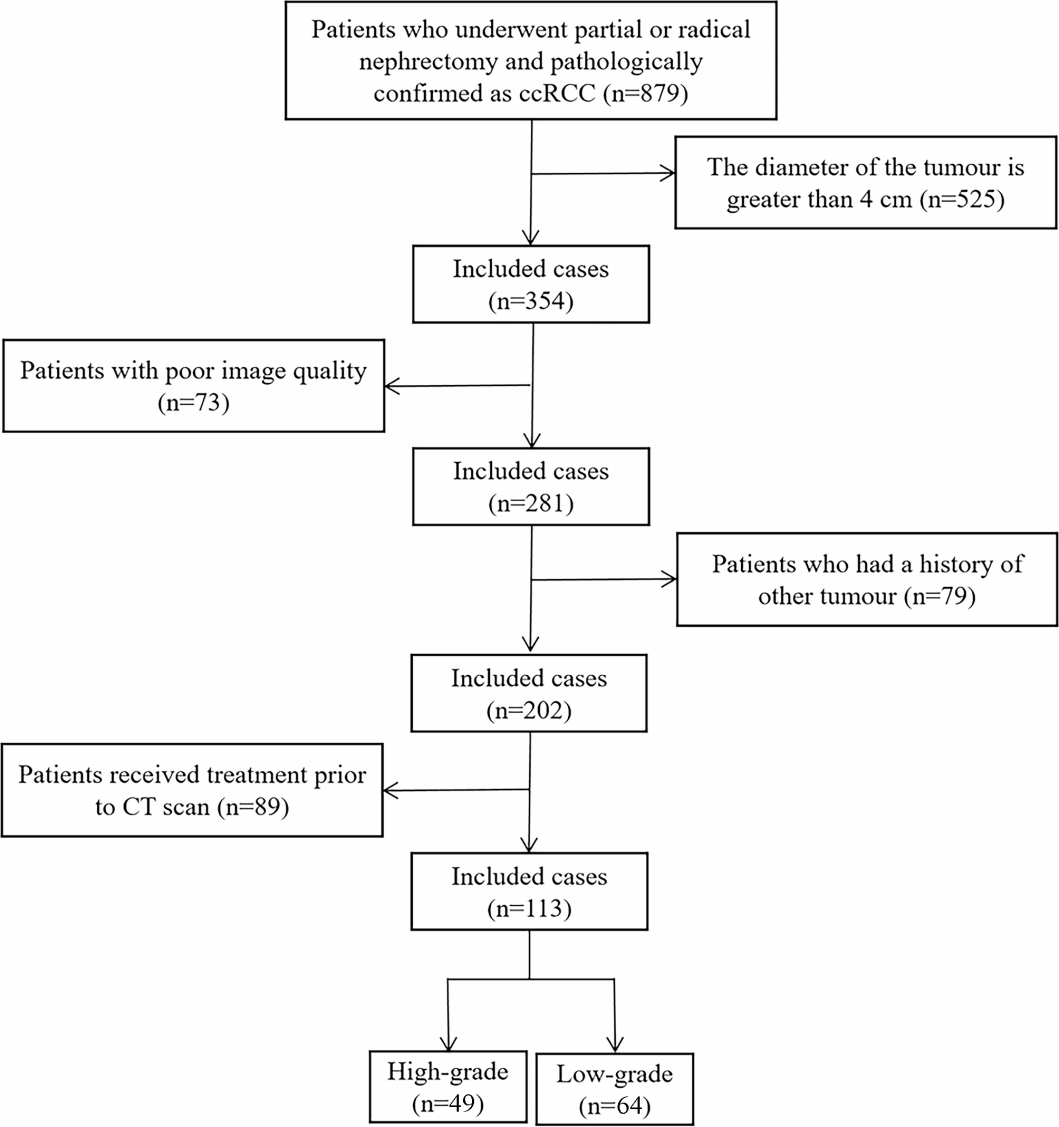
The flow chart of the patient recruitment

The Institutional Ethics Committee approved this retrospective study and waived the need for patient consent. The study included patients who underwent abdominal CT scans and were diagnosed with a renal tumor at our institution between January 2016 and January 2022. The inclusion criteria were as follows: (1) Patients with ccRCC who underwent a partial or radical nephrectomy. (2) Patients who had non-contrast and enhanced CT scans performed prior to surgery. (3) Patients with complete clinical information. The exclusion criteria were as follows: (1) Significant artefacts on CT images. (2) Tumors with a diameter greater than 4 cm. (3) Patients with a history of both kidney tumors and other tumors. (4) Patients who received treatment before the CT scan. A total of 113 patients were enrolled in the study, including 49 with high-grade small ccRCC and 64 with low-grade small ccRCC. In a ratio of 6:4, patients were randomly assigned to a training set (n = 67) and a testing set (n = 46). Figure 1 illustrates the workflow for enrolling the patient cohort.

### CT imaging acquisition

Routine clinical CT scans of the kidney are typically performed using 64-slice multidetector CT equipment. The CT scan parameters are as follows: the tube voltage is 120 kV-140 kV; tube current is 250mA-400 mA; slice thickness is 5 mm. Approximately 80 to 100 mL (1.5mL/kg) of contrast agents (Omnipaque, GE Healthcare) is injected into the antecubital vein at a rate of 3.0 mL/s using a high-pressure injector. Four phases of CT images are obtained: the unenhanced phase (UP), the corticomedullary phase (CMP) which is acquired 30 s after contrast injection, the nephrographic phase (NP) which is acquired 90 s after contrast injection, and the excretory phase (EP) which is acquired 180 s after contrast injection.

### Traditional radiological characteristics analysis

Two radiologists, Reader 1 and Reader 2, with 5 and 10 years of experience in diagnostic abdominal radiology, respectively, conducted a thorough review of the CT images. In cases where there was a disagreement between the two radiologists, they would engage in joint discussions to reach a consensus. The evaluation of the CT findings, including the maximum diameter of the tumor on axial CT images, shape (on axial slice), location, boundary, calcification, necrosis, renal vein invasion and lymph node metastasis, was performed by the radiologists without access to clinicopathologic information.

**Fig. 2:**
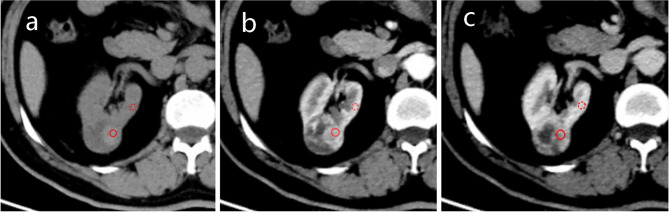
Selection of ROI and reference region. **a, b** and **c** correspond to the unenhanced phase (UP), corticomedullary phase (CMP), and nephrographic phase (NP). The red circle is one of three tumor ROI, selected from the parenchymal portion of the tumor where enhancement is evident. The red dotted circle is the reference region located in the cortical portion of the kidney. The ROI and reference region zones are in the same position in each scan phase

To determine the CT value of the tumor, a region of interest (ROI) was selected within the parenchyma of the tumor, excluding necrosis, calcification and vascularity. The ROIs in the study were chosen based on NP images as the tumor was clearly contrasted with the renal parenchyma in these images. Reader 1 selected three non-overlapping ROIs, took individual CT measurements for each, and then averaged the results. As CT scans are performed by different operators and on different patients, systematic inaccuracies in tumor CT value measurement may occur. To mitigate errors, the CT values of the cortex were measured in the cortical region of the kidney on the side of the tumor. Figure 2 shows an example of this method.

The average tumor attenuation value (TAV) for UP, CMP and NP was obtained from the ROI. The CT value measured in the renal cortex at each phase is known as the cortical attenuation value (CAV). The tumor enhancement value (TEV) and the cortex enhancement value (CEV) were calculated by subtracting the CT value of the UP: TEV_x_ = TAV_x_ – TAV_0_ and CEV_x_= CAV_x_ – CAV_0_, where x indicates the phase (x ranging from 1 to 2; 0 represent the UP, 1 represents the CMP, 2 represents the NP). To quantify the relative degree of enhancement within the tumor compared to the renal cortex, the ratio of TEV to CEV was calculated. This ratio is referred to as the relative enhancement value (REV), and it is represented as REV_x_ = TEV_x_/CEV_x_ [28].

### Construction of the clinical model

The differences between clinic-radiological characteristics of high-grade and low-grade small ccRCC were analyzed using univariate analysis. For categorical variables, the Chi-square test or Fisher exact test was used, while for continuous variables, the *t*-test or Mann-Whitney *U* test was applied. Statistically significant clinic-radiological characteristics were then used in a multivariate logistic regression analysis to identify the most valuable clinical factors and build a model. The odds ratio (OR) was calculated for each independent factor as a measure of relative risk prediction with a 95% confidence interval (CI).

### Tumor segmentation and extraction of radiomics features

**Fig. 3:**
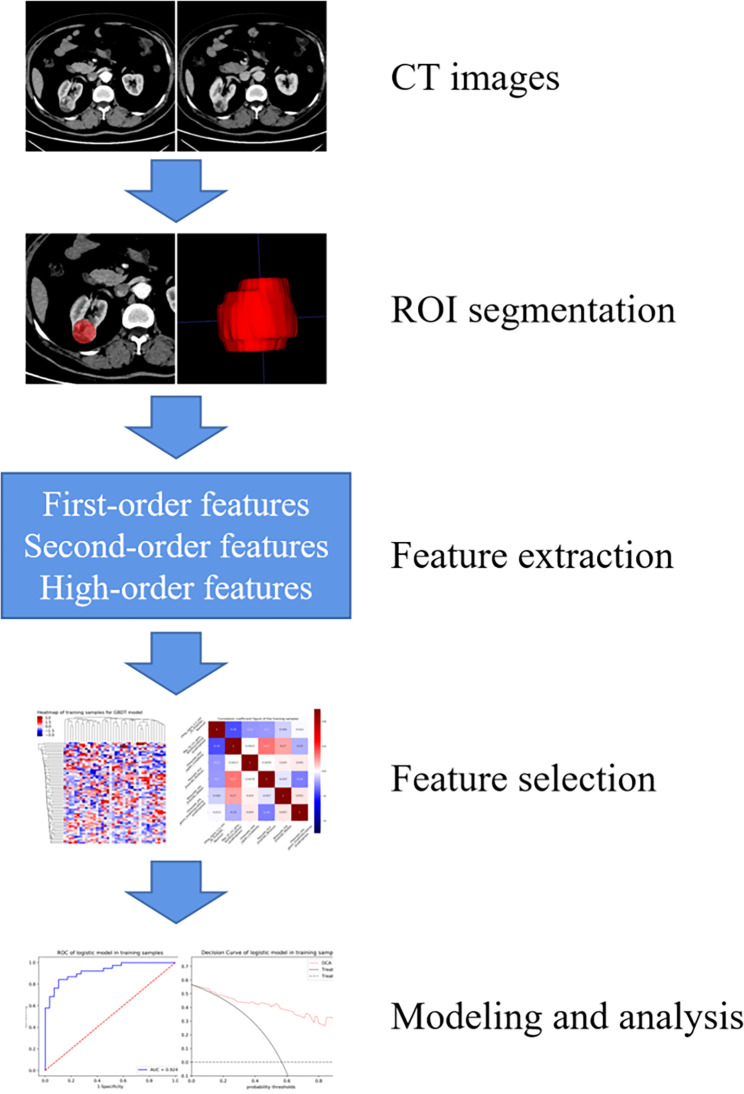
Schematic diagram of a radiomics study of renal tumors

**Fig. 4:**
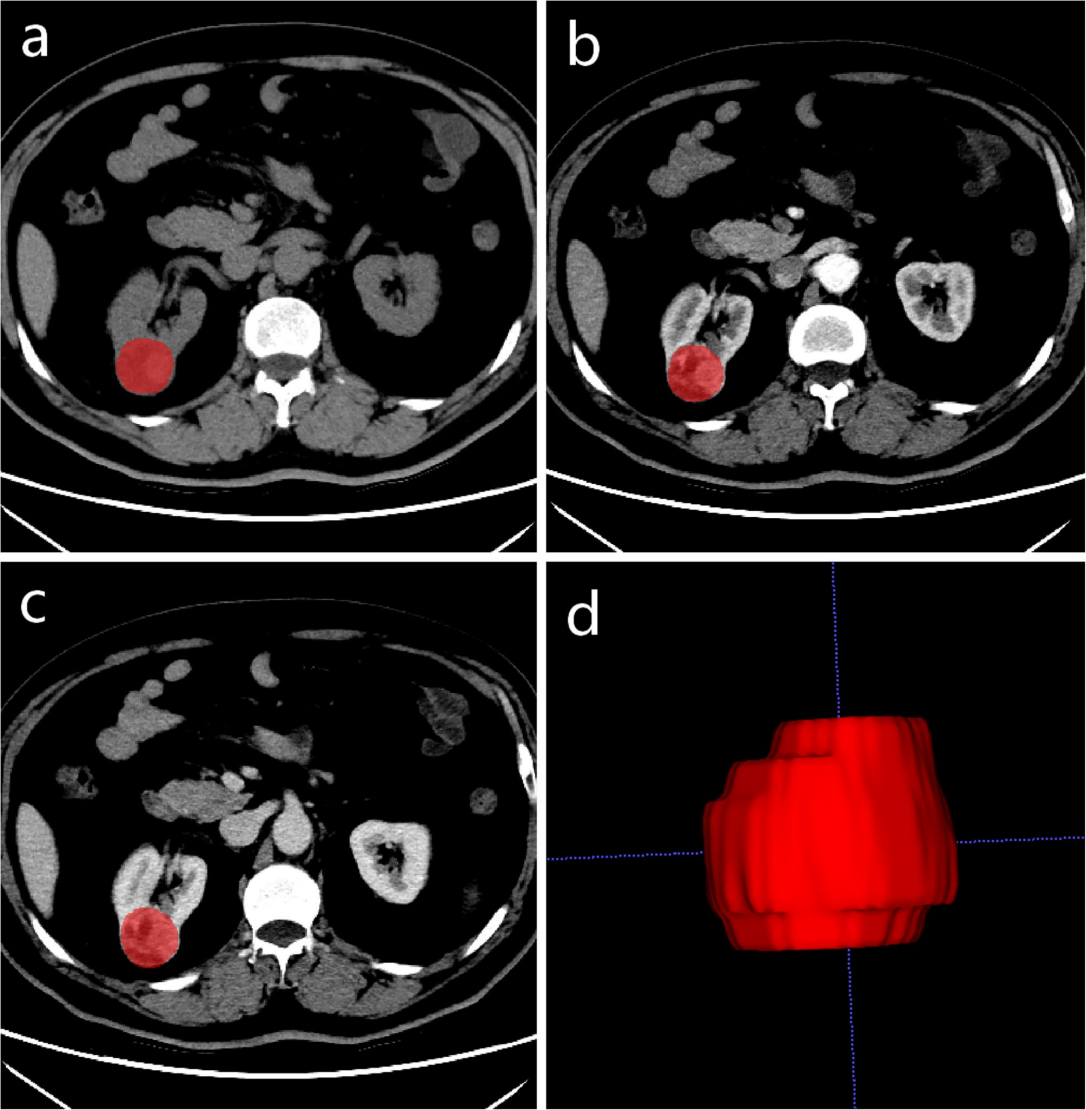
Manual three-dimensional (3D) of the tumor. a, b and c are the unenhanced phase (UP), the corticomedullary phase (CMP) and the nephrographic phase (NP), respectively. d is 3D volumetric reconstruction

Figure 3 illustrates the key steps in a radiomics model for renal tumors. The tumor’s volumes of interests (VOIs) were manually defined in ITK-SNAP software (version 3.8, www.itksnap.org) by two radiologists with extensive abdominal diagnostic experience (Fig. 4).

The extraction of features was performed using the Artificial Intelligence Kit software (A.K. Software, version 3.3.0.R). To minimize variability in the radiomics features, prior to extraction, the following image preprocessing techniques were applied: gray-level discretization, intensity normalization and voxel resampling. Subsequently, 1595 features were extracted from the UP, CMP and NP CT images by the open-source PyRadiomics library, respectively.

The intraclass correlation coefficient (ICC) was calculated to evaluate the consistency and reproducibility of the features. Features with ICC greater than 0.75 in both intra- and inter-observer agreement analyses were included in further analysis. Reader 1 and Reader 2 randomly segmented CT images of 20 patients (8 high-grade small ccRCC and 12 low-grade small ccRCC). Two weeks later, Reader 1 segmented these 20 patients once again.

### Construction of the radiomics model

To reduce redundant features and mitigate overfitting in the developed radiomics model, the following steps were undertaken for the features in the training set: (1) Features with an ICC greater than 0.75 were selected, (2) Univariate logistic analysis was conducted to identify the features that exhibited statistical significance, (3) The most significant features were chosen through a Gradient Boosting Decision Tree (GBDT) and further validated through multivariate logistic analysis, and (4) The remaining features were utilized to compute the radiomics score (Rad-score). Subsequently, a radiomics model was constructed in the training set using multivariate logistic regression. In order to assess the model’s performance, a separate radiomics model was built in the test set for testing purposes.

### Construction of radiomics nomogram and evaluation model performance

Clinical variables and Rad-score were combined to create a nomogram. Calibration curves were utilized to evaluate the calibration of the nomogram. The Hosmer-Lemeshow test was applied to assess the nomogram’s goodness of fit. The receiver operator characteristic (ROC) curves were utilized to evaluate the discrimination ability of the prediction model for high/low small ccRCC. The clinical validity of the clinical radiomics nomogram was further evaluated through decision curve analysis (DCA).

### Correlation between rad-score and immune infiltration

To assess the correlation between Rad-score and immune infiltration, we collected immunohistochemical images from 40 patients with small ccRCC and evaluated a total of 12 immunomarkers. Subsequently, hierarchical clustering analyses were performed for specific immunomarkers. We selected paraffin-embedded kidney tumor tissues from patients with one high-grade ccRCC and one low-grade ccRCC from our sample repository. Terminal deoxynucleotidyl transferase dUTP nick end labeling (TUNEL) assays were utilized to evaluate potential differences in apoptosis between low-grade and high-grade ccRCC [29].

### Statistical analysis

Python software (v.3.6.0) and R software (v.3.5.1) were used to perform the statistical analysis. A statistically significant difference between the two was defined as *p* < 0.05.

## Results

### Clinical characteristics and development of clinical model

The differences in clinical and radiological variables for the 113 patients are shown in Table 1. In 113 patients, Shape, TEV2, REV1 and REV2 were significantly different in high-grade ccRCC and low-grade ccRCC after univariate analysis (*p* < 0.05). After multivariate logistic regression analysis, shape (OR = 0.146, 95% CI = 0.027–0.790, *p* = 0.025) and REV2 (OR = 26.912, 95% CI = 1.389-521.396, *p* = 0.029) was an independent risk factor for identifying small ccRCC WHO/ISUP grade (Table 2).

**Table 1:** Clinic-radiological characteristics in training cohort and testing cohort

Clinic-radiological characteristics	Training cohort(n = 67)		*p*	Testing cohort(n = 46)		*p*
	High-grade	Low-grade		High-grade	Low-grade	
Gender			0.008			0.708
Male, n (%)	23(79%)	18(47%)		12(60%)	17	
Female, n (%)	6(21%)	20(53%)		8(40%)	9	
Age (years)	60(50–65)	55(48–67)	0.676	56(45–66)	55(43–64)	0.682
Maximum diameter (cm)	2.9(2.5–3.7)	3.0(2.7–3.3)	0.844	3.3(2.9–3.6)	3.1(2.3–3.5)	0.179
Shape (on axial slice)			0.007			0.012
Round, n (%)	20(69%)	36(95%)		12(60%)	24(92%)	
Not round, n (%)	9(31%)	2(5%)		8(40%)	2(8%)	
Location			0.570			0.074
Left, n (%)	14(48%)	21(55%)		13(65%)	10(38%)	
Right, n (%)	15(52%)	17(45%)		7(35%)	16(62%)	
Boundary			0.075			1.000
Clear, n (%)	20(69%)	33(87%)		17(85%)	23(88%)	
Blurred, n (%)	9(31%)	5(13%)		3(15%)	3(12%)	
Calcification			0.184			0.572
Present, n (%)	2(7%)	0(0%)		2(10%)	1(4%)	
Absent, n (%)	27(93%)	38(100%)		18(90%)	25(96%)	
Necrosis			0.952			0.085
Present, n (%)	17(59%)	22(58%)		15(75%)	13(50%)	
Absent, n (%)	12(41%)	16(42%)		5(25%)	13(50%)	
Renal vein invasion			1.000			1.000
Present, n (%)	0(0%)	1(3%)		0(0%)	0(0%)	
Absent, n (%)	29(100%)	37(97%)		20(100%)	26(100%)	
Lymph node metastasis			0.645			0.435
Present, n (%)	3(10%)	2(5%)		1(5%)	0(0%)	
Absent, n (%)	26(90%)	36(95%)		19(95%)	26(100%)	
TEV1 (HU)	80(59–101)	95(62–125)	0.215	100(76–124)	104(87–128)	0.444
TEV2 (HU)	71(58–95)	92(64–111)	0.027	84(59–104)	97(78–114)	0.086
REV1	0.76(0.59–0.95)	0.94(0.60–1.22)	0.034	0.91(0.57–1.09)	1.00(0.71–1.21)	0.231
REV2	0.61(0.47–0.70)	0.74(0.61–0.86)	0.003	0.67(0.51–0.84)	0.75(0.62–0.86)	0.465

*TEV*, tumor enhancement value; *REV*, relative enhancement value; 1, corticomedullary phase; 2, nephrographic phase

**Table 2:** Multivariate logistic regression analysis of the clinic-radiological characteristics in predicting the WHO/ISUP grade of small ccRCC.

Clinical characteristics	Multivariate analysis		
	OR	95% CI	*P* value
Shape	0.146	0.027–0.790	0.025
TEV2	1.001	0.965–1.037	0.976
REV1	1.473	0.122–17.812	0.761
REV2	26.912	1.389-521.396	0.029

*TEV*, tumor enhancement value; *REV*, relative enhancement value; 1, corticomedullary

phase; 2, nephrographic phase

### Extraction of features and filtering to create a radiomics model

**Fig. 5:**
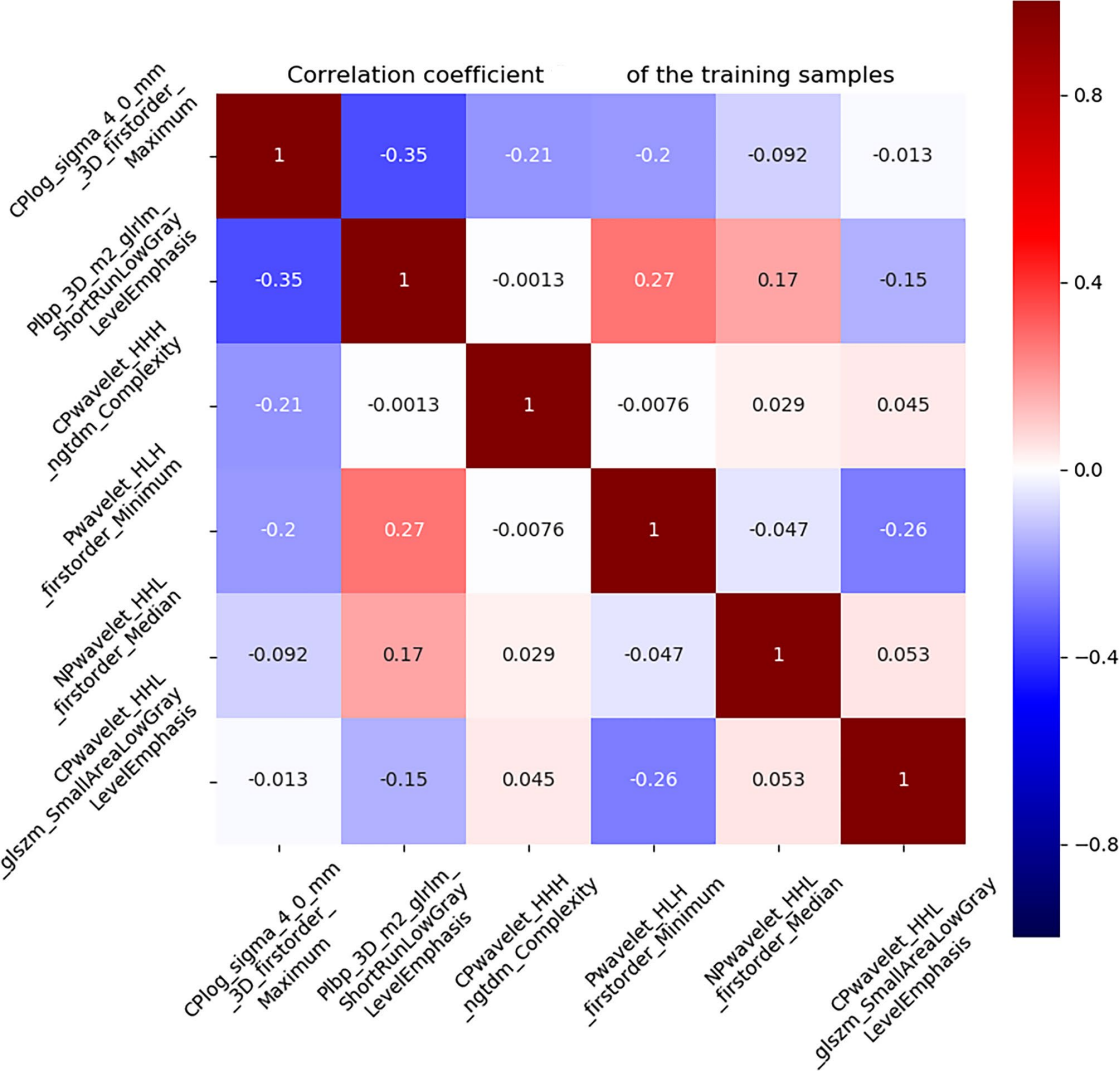
The correlation diagram of the six effective features screened out

Of the 4785 radiomics features in the three phases, 2560 had good repeatability (ICC > 0.75), and the dimensionality reduction section was based on these features. A total of 597 features were significantly different by univariate analysis. The six most valuable features were selected from 597 features based on the Gradient Boosting Decision Tree and multivariate logistic analyses (Fig. 5). These features were used to establish a radiomics model. The area under the curve (AUC) values in the training and testing sets are respectively 0.924 (95%CI, 0.868–0.969) and 0.869 (95%CI, 0.781–0.947) with the radiomics model. The Rad-score was calculated using six valuable features:

Rad-score = 0.9392–1.6715 × CMP-log_sigma_4_0_mm_3D_firstorder_Maximum.

+ 1.7051 × UP-lbp_3D_m2_glrlm_ShortRunLowGrayLeveIEmphasis.

– 1.0577 × CMP-wavelet_HHH_ngtdm_Complexity.

+ 1.6901 × UP-wavelet-HLH_firstorder_Minimum.

+ 1.2094 × NP-wavelet_HHL_firstorder_Median.

+ 0.9787 × CMP-wavelet_HHL_glszm_SmallAreaLowGrayLeveIEmphasis.

Figure S1 shows how the distribution of the Rad-score in the training and testing cohorts.

### The development of nomogram and evaluation of model performance

**Fig. 6:**
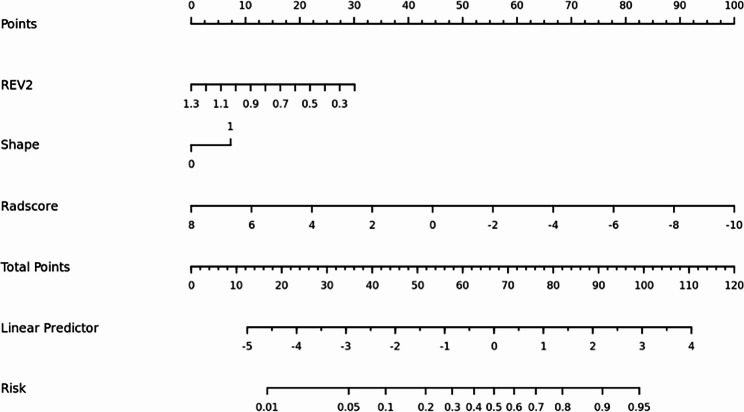
A radiomics nomogram distinguishing between high-grade and low-grade small ccRCC.

**Fig. 7:**
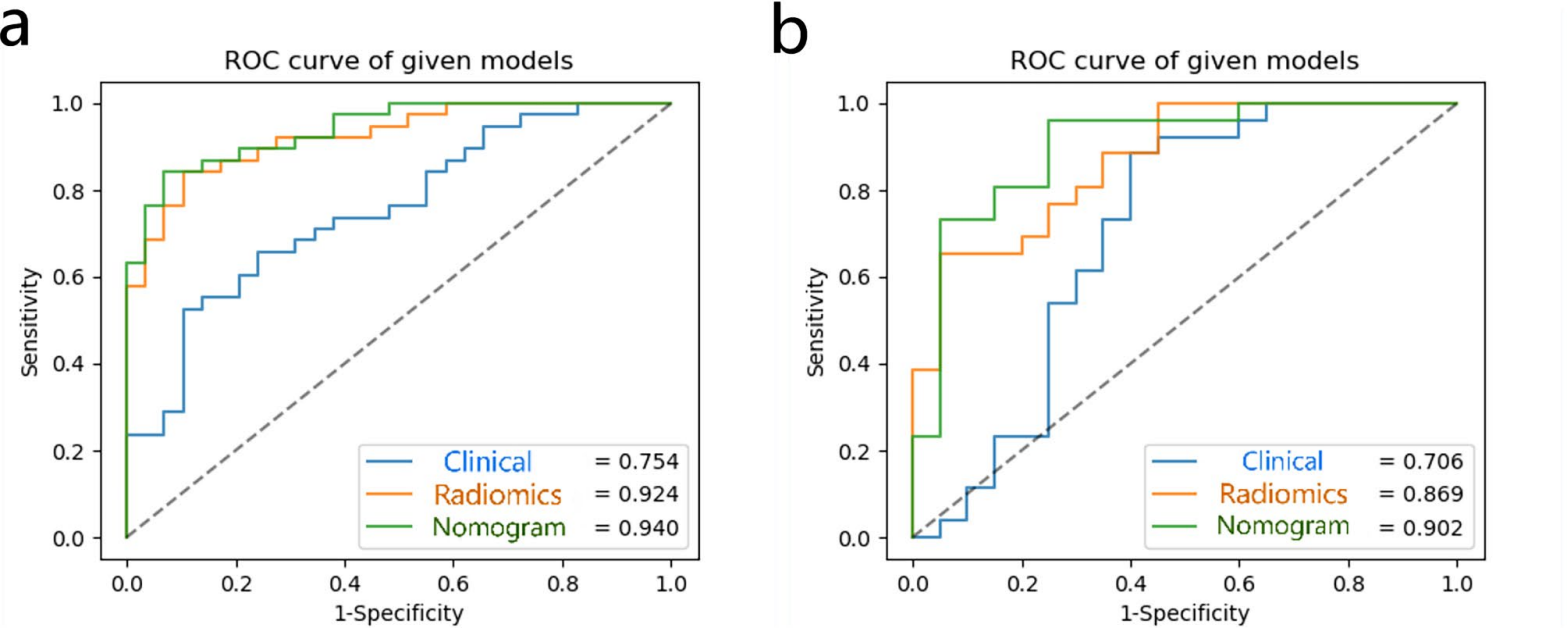
The ROC curves of the three models in the training (**a**) and testing (**b**) sets

**Fig. 8:**
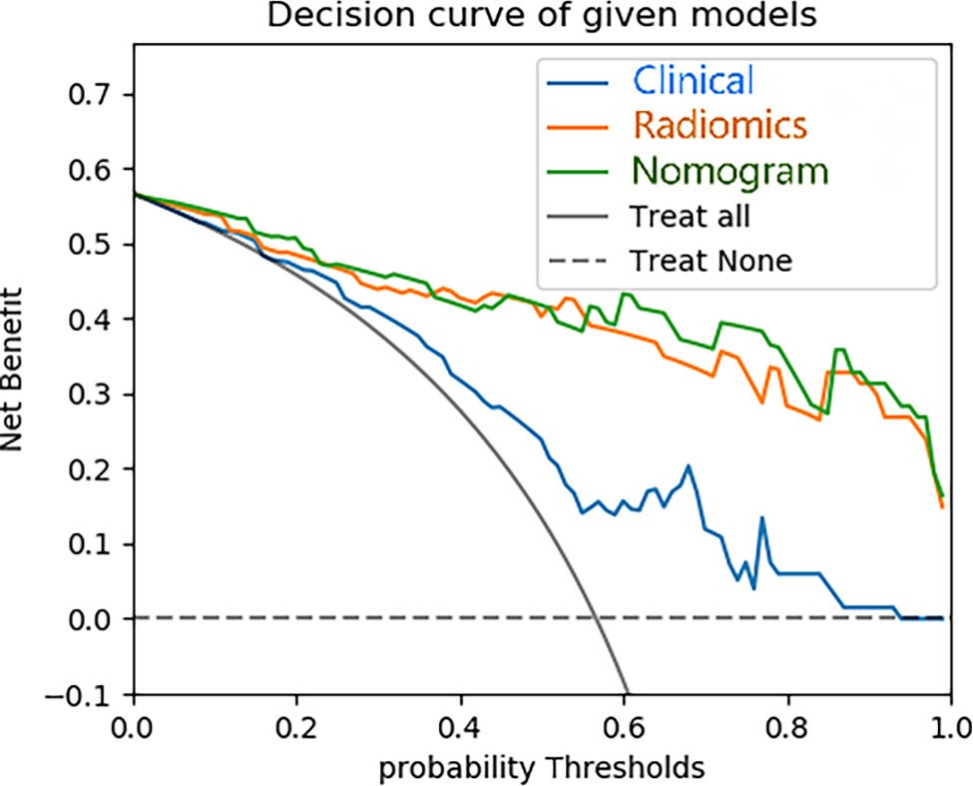
Decision curve analysis (DCA) for the clinical model, radiomics model and radiomics nomogram. The DCA indicated that more net benefits within the most of threshold probabilities were achieved using the radiomics nomogram

The clinic-radiological characteristics and Rad-score from the training cohort were subjected to a multivariate logistic regression analysis to obtain a radiomics nomogram score (Nomo-score): Nomo-score= -3.4699 + 0.9858 × Rad-score + 2.1449 × Shape + 2.4012 × REV2 (Fig. 6). The nomogram’s calibration curves in Figure S2 demonstrate the model’s strong clinical applicability. Table 3 shows the diagnostic effectiveness of the clinical model, the radiomics model and the radiomics nomogram. The ROC curves for the three models are shown in Fig. 7. The DCA curve illustrated in Fig. 8 showed the accuracy of the three models.

**Table 3:** Diagnostic performance of the clinical model, the radiomics model and the radiomics nomogram

Model	Training cohort				Testing cohort			
	AUC (95%CI)	Accuracy %	Specificity %	Sensitivity %	AUC (95%CI)	Accuracy %	Specificity %	Sensitivity %
Clinical model	0.754 (0.650–0.850)	67.2%	89.7%	50.0%	0.706 (0.554–0.845)	73.9%	60.0%	84.6%
Radiomics model	0.924 (0.871–0.968)	85.1%	89.7%	81.6%	0.869 (0.773–0.948)	76.1%	95.0%	61.5%
Radiomics nomogram	0.940 (0.894–0.977)	86.6%	93.1%	81.6%	0.902 (0.811–0.976)	84.8%	72.0%	92.3%

### The correlation between rad-score and the immune microenvironment

We examined the expression pattern and distribution of 12 immune markers in 40 patients with small ccRCC. To investigate the relationship between Rad-score and local immune status, we conducted and correlation analyses (Figure S3). We found that Rad-score was positively correlated with the expression of intratumoral CAIX (*p* = 0.043, R = 0.322), but negatively correlated with the expression of intratumoral Ki-67 (*p* = 0.026, R=-0.352). Terminal deoxynucleotidyl transferase dUTP nick end labeling assays performed on low-grade and high-grade kidney tumor sections showed that low-grade showed more apoptotic cells than high-grade (Figure S4).

## Discussion

With the increased detection rate of small ccRCC, the frequent underestimation of the histological grade of tumors on puncture biopsy, and the widespread use of active surveillance for patients with small RCC in clinical practice, a reliable method is needed to differentiate the histological grade of small ccRCC. This study has high accuracy in constructing a nomogram for the WHO/ISUP nuclear grading of small ccRCC based on clinic-radiological characteristics and radiomics features, with AUC values of 0.940 (95%CI, 0.894–0.977) and 0.902 (95%CI, 0.811–0.976) in the training and testing sets, respectively.

Previous studies have shown that the diagnostic of SRM is primarily based on imaging characteristics. Takahashi et al. [15] and Sasaguri et al. [14] demonstrated that CT images are highly effective in differentiating between benign and malignant SRM. Cohi et al. [16] found that CT imaging features could predict the histological grading of small ccRCC. In our study, four imaging features, shape, TEV2, REV1 and REV2, were significantly different in identifying high-grade from low-grade small ccRCC after univariate analysis. The results of Ding et al. [30] are consistent with our findings that high-grade ccRCC are more irregular in shape and high-grade ccRCC are less enhanced than low-grade ccRCC. We believe this is mainly because high-grade ccRCC are more malignant and more likely to invade surrounding tissues, making the shape irregular. High-grade ccRCC are prone to internal necrosis, and because of the active growth of the tumor tissue is more likely to block the blood vessels of the tumor, reducing the blood supply to the tumor. However, Halefoglu et al. [31] showed that high-grade RCC tumor enhancement values were higher than low-grade RCC, which is inconsistent with our results. We believe that this discrepancy is mainly due to selection bias in choosing the sample for the study; we studied only one subtype, ccRCC, whereas Halefoglu et al. chose both ccRCC and papillary renal cell carcinoma (pRCC). Some studies have shown that necrosis can be used as an essential risk factor to differentiate the histological grade of tumors. But in this research, necrosis was not significantly different between the two groups of tumors, which in our analysis may be because the sample selected was all < 4 cm [31, 32].

As a new form of artificial intelligence, radiomics is widely used in the diagnostic and differential diagnosis of SRMs [22, 23, 25, 26, 33]. It can extract information from medical images that the human eye cannot see. Feng et al. [23] developed a machine learning model for differentiating angiomyolipoma without visible fat (AMLwvf) and RCC based on 58 patients with SRMs, with accuracy, sensitivity, specificity and AUC of 93.9%, 87.8%, 100% and 0.955, respectively. Yang et al. [25] collected 163 patients with SRM, including 118 RCC and 45 AMLwvf, and the final classification model was constructed with an AUC of 0.9. These studies were mainly based on SRM for benign-malignant discrimination and extracted features based only on the largest dimension of the tumor and did not include the full 3D-ROI, thus not containing the complete information of the tumor. Hagi-Momenian et al. [24] constructed various machine learning models based on noncontrast phase, CMP and NP for histological grading and tumor subtyping of small pRCC, respectively. The models constructed based on the features extracted from CMP had the highest AUC values of 0.97-1.0. Haji-Momenian et al. [33] extracted six histogram features and 31 texture features from the noncontrast phase, CMP and NP images of small ccRCC patients. The analysis revealed no significant difference between the features extracted from the noncontrast phase and NP in high-grade and low-grade small ccRCC. Twenty-three features extracted from CMP were significantly different, and multiple machine learning models were built based on these features with a maximum AUC value of 0.97. This is in line with our findings, where we extracted a total of 4785 features from UP, CMP and NP, and filtered them to obtain six valid features, three of which were from CMP. The results suggest that CMP images are valuable in distinguishing the histological grading of small RCC. Previous studies extracted only a few dozen features from the images, which hardly reflect the actual information of the tumor, and thousands of features were extracted for the analysis in this study. Previous studies have focused only on models built through radiomics features, ignoring the information contained in the medical images themselves. The AUC value of the nomogram constructed by combining the radiomics features with the clinical characteristics was 0.940 in the training set and 0.902 in the testing set, which were both higher than those of the clinical model and the radiomics model. In this study, we observed that a lower Rad-score value is associated with a higher likelihood of high-grade small ccRCC and a worse prognosis. Additionally, Rad-score showed a positive correlation with CAIX and a negative correlation with Ki-67. This suggests that lower expression of CAIX and higher expression of Ki-67 are associated with a higher likelihood of high-grade ccRCC and a worse prognosis, which is consistent with the findings of previous studies [34, 35]. TUNEL assays revealed variations in apoptosis among different grades of ccRCC, with a higher percentage of apoptosis observed in tumors with lower malignancy levels. This finding aligns with prior research studies [36, 37].

Our study has several limitations. Firstly, the sample size for this study is small, and there is a lack of external validation data. This is due to the fact that we only included ccRCC tumors with a diameter less than 4 cm, and more cases could be collected prospectively for future studies. Secondly, the tumor segmentation in this study was based on manual segmentation, which is both time-consuming and subjective. It would be beneficial to investigate an automated segmentation method for kidney tumors in the future. Lastly, this study only classified ccRCC as high-grade and low grades, which is not a highly accurate classification system. Future studies could develop a model with four categories.

## Conclusion

We have developed and validated a CT-based radiomics nomogram that incorporates a rad-score, shape and REV2 to predict the grading of small ccRCC preoperatively. This nomogram will assist clinicians in making informed diagnostic and treatment decisions.

### Electronic supplementary material

Below is the link to the electronic supplementary material.

Supplementary Material 1

## Data Availability

The datasets used and/or analyzed during the current study are available from the corresponding author on reasonable request.
