# Supplementary material for: Multiphase CT radiomics nomogram for preoperatively predicting the WHO/ISUP nuclear grade of small (< 4 cm) clear cell renal cell carcinoma
Source: BMC Cancer. 2023 Oct 9;23:953. doi: 10.1186/s12885-023-11454-5 (PMC10561466; doi:10.1186/s12885-023-11454-5)
Supplement: Supplementary file 1 — Supplementary Material 1 [file 12885_2023_11454_MOESM1_ESM.docx]

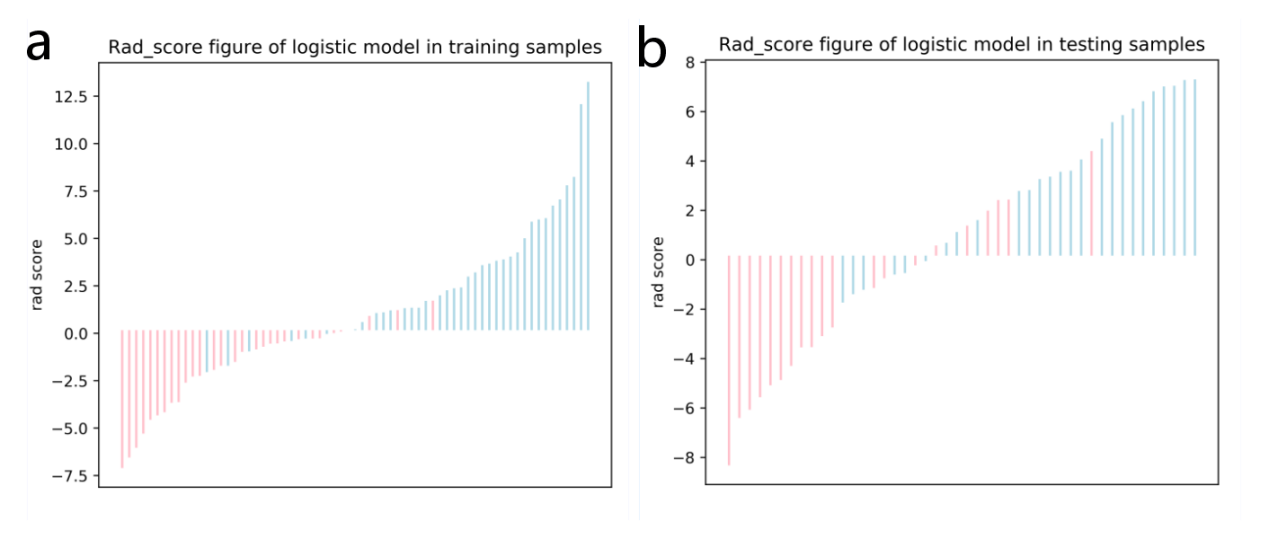


**Figure S1**. The Rad-score for each patient in the training (a) and testing (b) sets.


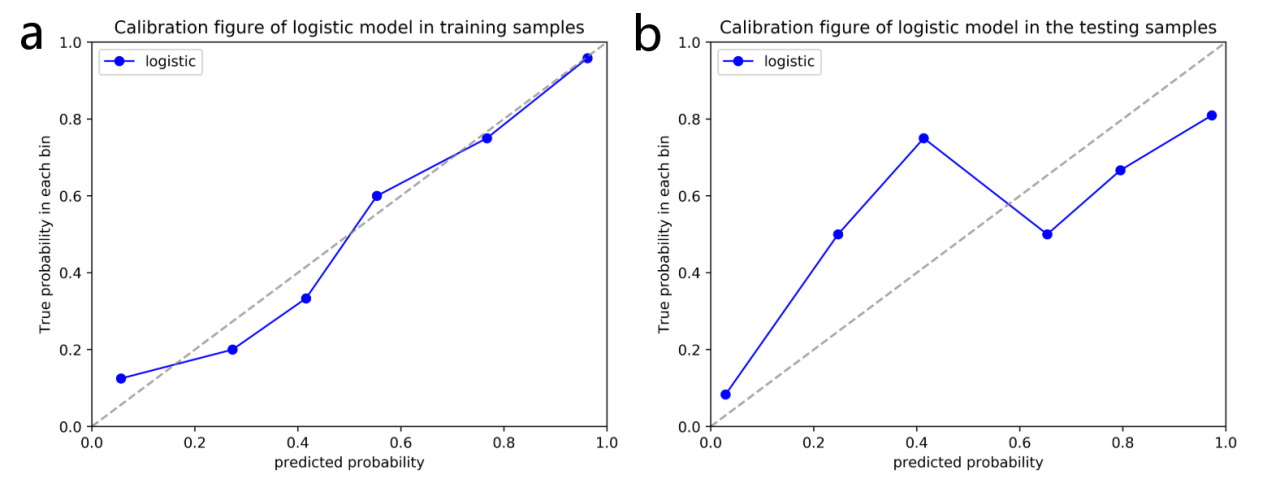


**Figure S2.** Radiomics nomogram calibration curves for the training (a) and testing (b) sets. The calibration curves show a good fit for the nomogram. The 45° straight lines indicated a perfect match between the true (Y-axis) and predicted (X-axis) probabilities. The closer the distance between the two curves, the better accuracy.

**
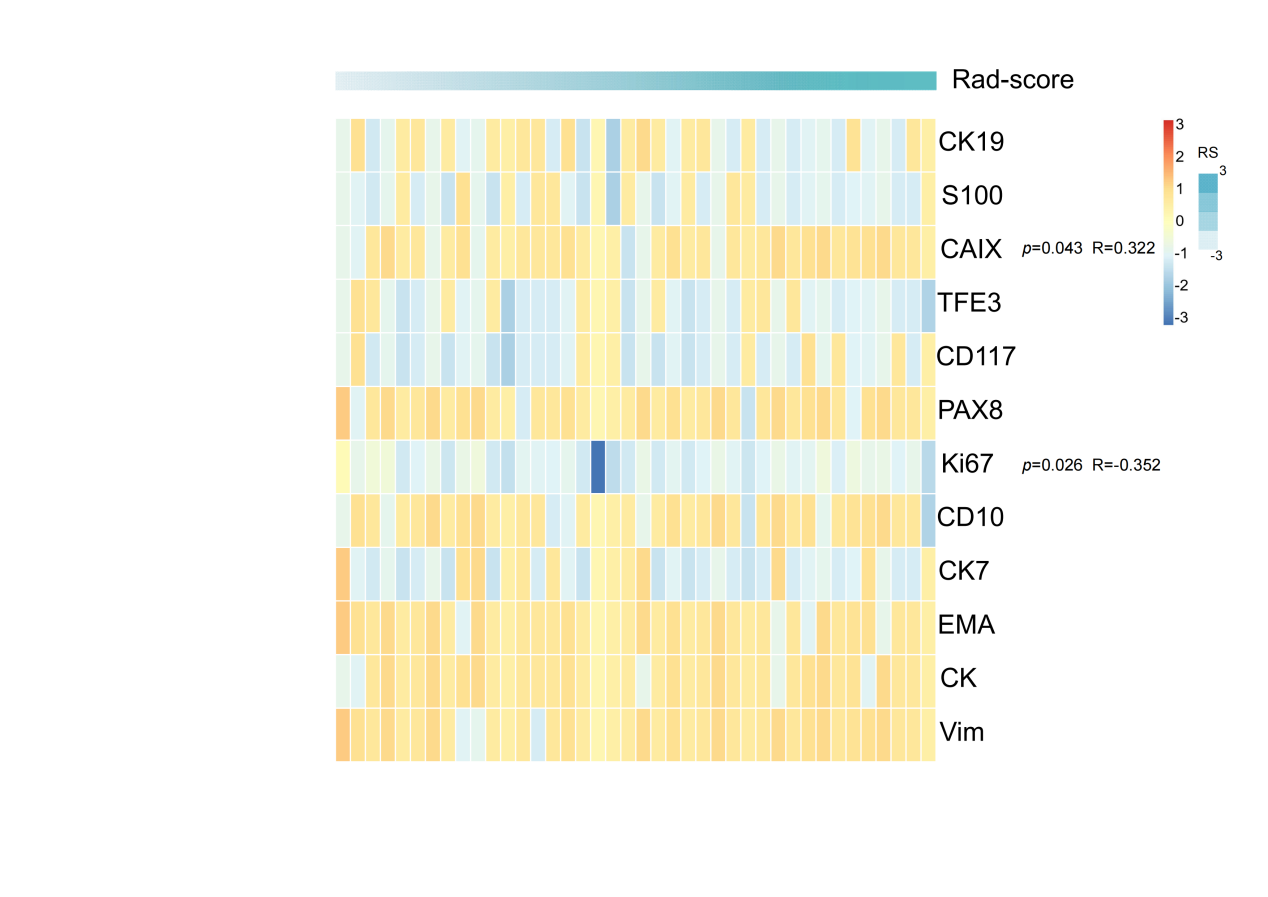
**

**Figure S3.** Heatmap and cluster analysis of immunohistochemistry with Rad-score.

**
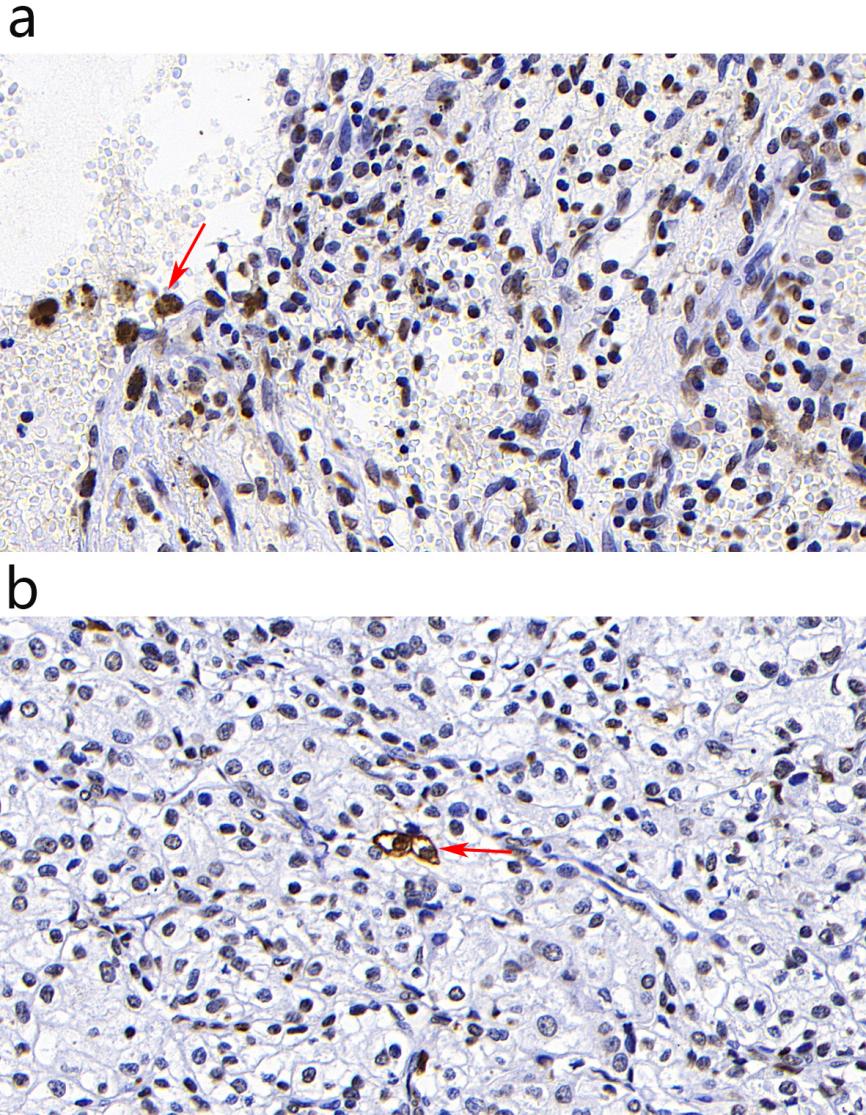
**

**Figure S4.** TUNEL assays were conducted on both low-grade and high-grade ccRCC samples: a) one patient with ccRCC graded as grade I by WHO/ISUP. b) one patient with ccRCC graded as grade IV by WHO/ISUP. The red arrows highlight apoptotic cells.
